# Maternal Glycemia and Its Pattern Associated with Offspring Neurobehavioral Development: A Chinese Birth Cohort Study

**DOI:** 10.3390/nu17020257

**Published:** 2025-01-11

**Authors:** Zhichao Yuan, Tao Su, Li Yang, Lei Xi, Hai-Jun Wang, Yuelong Ji

**Affiliations:** 1 Department of Maternal and Child Health, School of Public Health, Peking University, Beijing 100191, China; 2 Tongzhou Maternal and Child Health Care Hospital of Beijing, Beijing 101101, China

**Keywords:** maternal glycemia, neurobehavior development, pregnancy stages, TyG index

## Abstract

Background/Objectives: This study investigates the impact of maternal glycemic levels during early and late pregnancy on offspring neurodevelopment in China. Methods: Fasting plasma glucose (FPG) and triglyceride (TG) levels were measured in maternal blood during pregnancy, and the TyG index was calculated to assess insulin resistance. Hyperglycemia was defined as FPG > 5.1 mmol/L. Neurodevelopmental outcomes in offspring aged 6–36 months were evaluated using the China Developmental Scale for Children, focusing on developmental delay (DD) and developmental quotient (DQ). Mothers were categorized into four glycemic groups: healthy glycemia group (HGG), early pregnancy hyperglycemia group (EHG), late pregnancy hyperglycemia group (LHG), and full-term hyperglycemia group (FHG). Linear and logistic regression models were applied. Results: Among 1888 mother–child pairs, hyperglycemia and FPG were associated with an increased risk of overall DD (aOR = 1.68; 95% CI 1.07–2.64) and lower DQ (aBeta = −1.53; 95% CI −2.70 to −0.36). Elevated FPG was linked to DD in fine motor and social behaviors. Compared to HGG, LHG and FHG significantly increased the risk of overall DD (aOR = 2.18; 95% CI 1.26–3.77; aOR = 2.64; 95% CI 1.38–5.05), whereas EHG did not. Male offspring were particularly vulnerable to early pregnancy hyperglycemia (aBeta = −2.80; 95% CI −4.36 to −1.34; aOR = 2.05; 95% CI 1.10–3.80). Conclusions: Maternal glycemic levels during pregnancy influence offspring neurodevelopment, with persistent hyperglycemia significantly increasing DD risk. Early pregnancy hyperglycemia particularly affects male offspring, underscoring the need for glycemic management during pregnancy.

## 1. Introduction

The newborn brain is not fully mature at birth. The first three years of life are critical for neurodevelopment. A newborn’s total cerebrum volume constitutes merely 25% of an adult’s brain volume [1]. By the age of three, this volume rapidly increases to around 80% of the adult volume [1]. Previous studies have indicated that during this period, brain tissue dramatically shifts from predominantly white matter to gray matter [2,3]. This differentiation in brain tissue likely reflects underlying changes in myelination and synaptic proliferation. Neurodevelopment is primarily assessed through five domains of cognition and behavior in children: gross motor, fine motor, cognitive ability, language ability, and social behavior [4]. Numerous studies have reported close associations between neurodevelopmental delay and subsequent neurodevelopmental disorders such as autism, language disorders, and developmental motor coordination disorders [5,6,7]. Several studies have also indicated that children’s neurodevelopment is influenced by maternal conditions such as obesity, maternal immune activation, maternal nutrition, viral infections, and preterm birth [8,9,10,11,12]. However, these current hypotheses do not fully explain the origins of neurodevelopmental delay. Therefore, it is essential to further investigate novel potential risk factors for neurodevelopment.

Blood glucose is a crucial source of energy for fetal growth and development in utero. Some studies have suggested that maternal glycemia during pregnancy is associated with adverse neurodevelopmental outcomes in offspring during early life. For instance, a Chinese study indicated that maternal gestational diabetes mellitus (GDM) increased the risk of communication delay at 12 months of age [13]. Similarly, research from southern Israel reported associations between GDM and a range of neurodevelopmental disorders, including autism, eating disorders, obstructive sleep apnea, epilepsy, and cerebral palsy [14]. Conversely, a large-scale cohort study in the United States found that GDM was not a significant risk factor for autism spectrum disorder (ASD). These discrepancies may be due to differences in population ethnicity, study design, and variability in covariables.

Glycemic patterns during pregnancy, reflecting fluctuations and trends in blood glucose levels, are shaped by physiological changes that support fetal growth [15]. While maternal glucose levels often vary across trimesters, significant deviations from typical glycemic patterns can influence fetal growth and neurodevelopment. Abnormal glycemic patterns, such as prolonged hyperglycemia, may adversely impact neurodevelopment by altering the intrauterine environment [16]. These alterations can disrupt key neurodevelopmental processes, potentially increasing the risk of cognitive, motor, and behavioral delays in offspring [17]. Despite the relevance of glycemic patterns, limited research has examined how these patterns, beyond just gestational diabetes, may impact neurodevelopmental outcomes. Existing studies predominantly focus on the impact of blood glucose levels at a single time point on maternal and fetal health, whereas the effects of blood glucose fluctuations across multiple time points remain largely unexplored.

Previous studies have shown that maternal insulin resistance (IR) increases as pregnancy progresses [18]. This rise in IR is primarily driven by placental hormones, which work to reduce maternal insulin sensitivity and enhance glucose availability in the later stages of pregnancy [19]. While this process is generally beneficial, abnormal increases in IR or poor regulation of glucose can lead to hyperglycemia, potentially affecting fetal development. Despite its importance, few studies have examined the effects of maternal IR on fetal neurodevelopment.

In this study, we evaluate the associations between glycemic indicators and neurodevelopment in offspring. By examining broader glycemic patterns across both early and late pregnancy, we aim to assess how variations in maternal glucose levels may influence neurodevelopmental outcomes in offspring. Additionally, we calculate the Triglyceride–Glucose (TyG) index, an integrated biomarker combining fasting plasma glucose and triglycerides, to investigate the relationship between IR and neurodevelopment across different pregnancy stages.

## 2. Materials and Methods

### 2.1. Study Design and Participants

**Figure 1:**
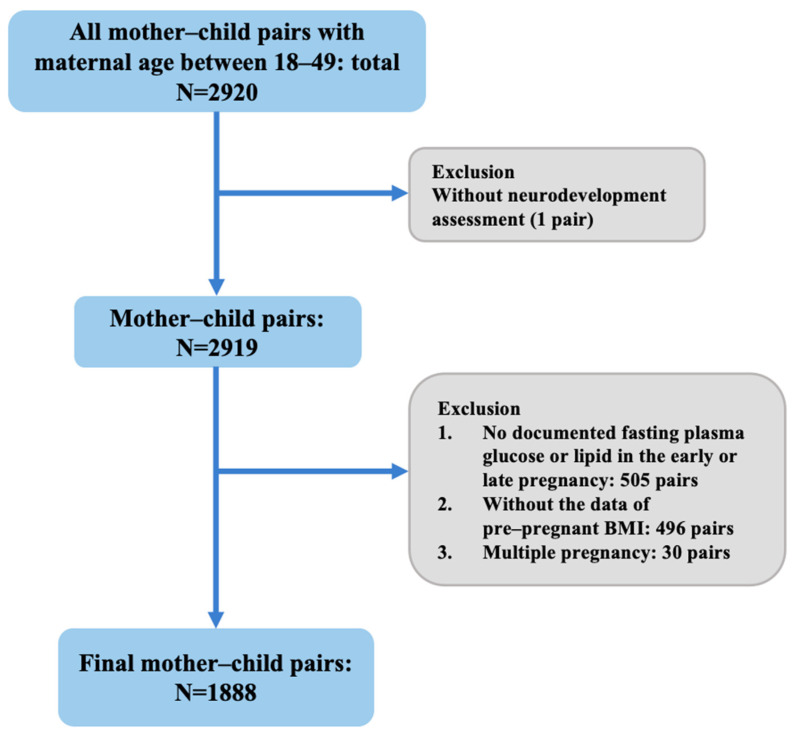
Study flowchart. Flowchart illustrating the selection of the mother–child pairs in current study.

This study was conducted using the Peking University Retrospective Birth Cohort in Tongzhou based on the hospital information system. To protect participant privacy, study data were extracted without personal identifiable information. Pregnant women who gave birth between January 2013 and December 2020 and received child health care examination for their children at Tongzhou Maternal and Child Health Care Hospital of Beijing were included for this study. Sociodemographic and health details were recorded during antenatal examinations and at delivery, including maternal age (continuous), educational levels (≤12 years vs. >12 years), occupation (blue-collar vs. white-collar vs. freelancer), parity (primiparity vs. multiparity), body mass index (BMI) categories (<25 kg/m^2^ vs. ≥25 kg/m^2^), folate intake (regular vs. irregular), season of conception (spring vs. summer vs. autumn vs. winter), sex of the infant (male vs. female), gestational age (continuous), birthweight (continuous), and age of offspring (continuous). Initially, 2920 pairs of mothers and children with maternal ages ranging from 18 to 49 years were recruited. The exclusion criteria for this study were as follows: (1) multiple pregnancies, (2) lack of documented fasting plasma glucose (FPG) or triglyceride (TG) measurements in the early and late pregnancy, (3) missing pre-pregnancy BMI data, (4) lack of neurodevelopment assessment. Finally, 1888 mother–child pairs completed examinations of blood glucose and lipids during the early and late pregnancy. The flowchart showing the participant selection process is presented in Figure 1. This study was approved by the Institutional Review Board of Peking University Health Science Center (IRB00001052-21023, approval date: 1 March 2021).

### 2.2. Maternal Glycemia Indicators

Fasting venous blood samples from the pregnancies were collected by specialist medical staff during the early and late pregnancy antenatal visits. Early pregnancy was defined as a gestational age of less than 13 weeks; otherwise, it was defined as late pregnancy. FPG and TG were tested using an automatic hematology analyzer in the clinical lab at the Tongzhou Maternal and Child Health Care Hospital of Beijing. The TyG index was calculated as Ln[TG (mg/dL) × FPG (mg/dL)/2] [20]. Hyperglycemia was defined as an FPG above 5.1 mmol/L according to the criteria of the International Association of Diabetes and Pregnancy Study Group (IADPSG) [21].

### 2.3. Neurodevelopmental Assessment

The neurodevelopment of the children, assessed between 6 and 36 months of age, was evaluated by professional childcare physicians using the China Developmental Scale for Children (CDSC) [22]. The CDSC is an indigenous developmental assessment tool designed and validated for the developmental stages of Chinese children since the early 1980s [23]. The five subscales of the CDSC (gross motor, fine motor, cognitive ability, language ability, and social behavior) are consistent with the relevant subscales in the Gesell Developmental Schedules. In this study, all children were thoroughly assessed on these five subscales, with the development quotient (DQ) calculated for each. The DQ is calculated as the developmental age divided by the chronological age, then multiplied by 100. Overall neurodevelopment was assessed using the mean values of the five subscale DQs. Neurodevelopmental delay was defined as overall DQ of less than 80.

### 2.4. Statistical Analysis

Descriptive analyses were conducted to compare the characteristics of mother–child pairs between those with normal development (ND) and those with developmental delay (DD). Continuous variables are presented as mean values with standard deviations (SD), and categorical variables are shown as frequencies (percentages).

In the primary analysis, we investigated the associations between glycometabolic indicators during the entire pregnancy (including FPG, TyG index, and hyperglycemic status) and overall neurodevelopment, as well as five domains: gross motor, fine motor, cognitive ability, language ability, and social behavior. The outcome variables included continuous development quotient and dichotomized development status. Linear and logistic regression models were used to estimate regression coefficients (Beta), odds ratio (OR), and 95% confidence intervals (95% CIs). The adjusted model, additionally adjusted for multiple covariates and calculated the adjusted regression coefficients (aBeta), adjusted odds ratio (aOR), and 95% CI to evaluate the relationship between glycometabolic indicators during pregnancy and offspring neurodevelopment. The covariates in the adjusted model included maternal age, educational level, occupation, parity, pre-pregnancy BMI, folate intake, season of conception, sex of the offspring, gestational age, birthweight, and age of the offspring. In the secondary analysis, linear and logistic regression models were rerun to examine the stage-specific effects during early and late pregnancy.

The study further analyzed the association between glycemic patterns during pregnancy and offspring neurobehavioral development. Glycemic patterns among pregnant women were classified into four distinct categories based on the hyperglycemic status during early and late pregnancy: (1) the healthy glycemia group (HGG), where glucose levels were within the healthy range in both early and late pregnancy; (2) the early pregnancy hyperglycemia group (EHG), characterized by hyperglycemia in early pregnancy and normoglycemia in late pregnancy; (3) the late pregnancy hyperglycemia group (LHG), with normoglycemia in early pregnancy and hyperglycemia in late pregnancy; and (4) the full-term hyperglycemia group (FHG), which exhibited hyperglycemia throughout both early and late pregnancy. Utilizing a logistic regression model and setting the healthy glycemia group as the reference, we evaluated the relationship between these glycemic pattern categories and the neurobehavioral development of the offspring.

Subgroup analyses were performed to test the effects of maternal glycemia on neurodevelopment across different strata of the offspring’s sex, and the interaction effects of glycemic indicators with sex were assessed. Given the close association between maternal age and folate intake with infant neurodevelopment, we conducted two sensitivity analyses: one for women under 35 years of age and another for women with regular folate intake. A two-sided *p* value of less than 0.05 was considered to indicate a significant difference, while a two-sided *p* value of less than 0.10 was defined as marginally significant due to the limited sample size.

## 3. Results

### 3.1. Participant Characteristics

In this study, 1888 mother–child pairs were included. The proportion of DD was 9.0% (170 out of 1888). The characteristics of the mothers and offspring are presented in Table 1. The mean maternal age was 28.2 years (SD 3.7). A higher percentage of DD was observed among mothers with lower education levels, those working in white-collar occupations, and those with multiparity. The mean age of the offspring was 10.1 months (SD 6.1). Offspring with DD tended to have lower birth weights and smaller gestational ages. Maternal and offspring characteristics stratified by hyperglycemia status are summarized in Table S1. In addition, the characteristics of glycemic indicators in early and late pregnancy are summarized in Table S2, and the DQ for overall development and specific domains is presented in Table S3.

### 3.2. Associations Between Full-Term Glycemic Indicators and Neurobehavioral Outcomes

The association between maternal glycometabolic levels throughout the entire pregnancy and offspring neurobehavioral development is shown in Table 2. After adjusting for covariates, higher FPG levels were associated with a decrease in the overall DQ (aBeta = −0.97, 95%CI −1.67 to −0.28) and an increased risk of overall DD (aOR = 1.37, 95% CI 1.07–1.76). Similarly, compared to the normoglycemic group, offspring in the hyperglycemic group (FPG > 5.1 mmol/L) showed a significantly lower overall DQ (aBeta = −1.13, 95% CI: −2.05 to −0.21) and a higher risk of DD (aOR = 1.68, 95% CI: 1.07 to 2.64). The TyG index was also correlated with overall development, showing an association with a lower DQ (aBeta = −1.53, 95% CI: −2.70 to −0.36) and an increased risk of DD (aOR = 1.69, 95% CI: 0.97 to 2.93). Regarding specific developmental domains, both FPG levels and hyperglycemia were found to be associated with fine motor, language ability, and social behavior domains. The unadjusted relationships between glycemic indicators and neurodevelopmental outcomes are summarized in Table S4.

### 3.3. Impact of Early and Late Pregnancy Glycemic Indicators on Neurobehavioral Development in Offspring

**Figure 2:**
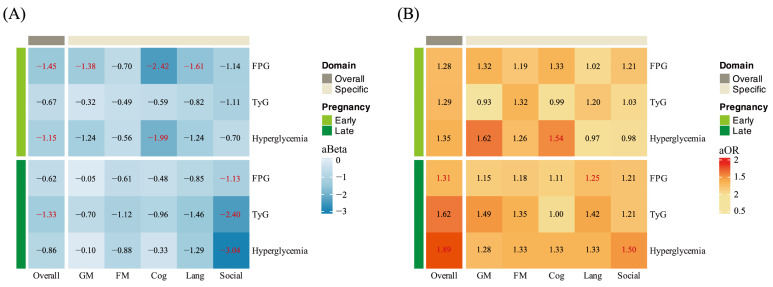
The associations between glycemia indicators across multiple pregnant stages and neurodevelopment in offspring. (**A**) illustrates the relationships between glycemic indicators and developmental quotient (DQ) in overall development as well as in specific domains. (**B**) displays the associations between glycemic indicators and the risk of developmental delay (DD). Significant associations are highlighted in red.

Several significant associations between glycemic indicators in early and late pregnancy and DQ were observed across overall and specific developmental domains (Figure 2A). In early pregnancy, FPG levels and hyperglycemia were negatively correlated with DQs in overall (aBeta = −1.45, 95% CI −2.58 to −0.33; aBeta = −1.15, 95% CI −2.22 to −0.07, respectively) and cognitive development (aBeta = −2.42, 95% CI −4.19 to −0.65 and aBeta = −1.99, 95% CI −3.68 to −0.30, respectively). FPG levels in early pregnancy were also associated with lower DQ of gross motor and language ability. In late pregnancy, TyG index was observed to correlate with the DQ in overall development and social behavior (aBeta = −1.33, 95% CI −2.46 to −0.19 and aBeta = −2.40, 95% CI −4.21 to −0.58). Furthermore, there were significant negative associations between FPG, hyperglycemia, and DQ in social behavior. Figure 2B showed the impact of glycemic indicators in early and late pregnancy on the risk of DD across overall and specific developmental domains. In early pregnancy, hyperglycemia increased the risk of DD in cognitive ability (aOR = 1.54, 95% CI 1.08–2.18). In late pregnancy, significant associations between FPG and risk of DD in overall and language ability (aOR = 1.31, 95% CI 1.03–1.66; aOR = 1.25 95% CI 1.00–1.55). Additionally, hyperglycemia in late pregnancy was associated with an increased risk of delay in overall development and social behavior (aOR = 1.89, 95% CI: 1.18–3.02; aOR = 1.50, 95% CI: 1.04–2.18). The unadjusted associations between glycemic indicators in early and late pregnancy and neurodevelopmental outcomes are presented in Tables S5 and S6.

### 3.4. Associations Between Glycemic Patterns and Neurobehavioral Outcomes

Compared to the HGG, both the LHG and FHG showed decreased DQ and an increased risk of DD in overall development (all *p* < 0.01, Table 3). Regarding specific developmental domains, LHG was associated with lower DQs in fine motor, language ability, and social behavior (aBeta ranging from −1.62 to −3.88). Additionally, compared with the HGG, the LHG consistently showed positive associations with an increased risk of DD in fine motor and social behavior (aOR = 1.62, 95% CI: 1.03–2.54; aOR = 1.68, 95% CI: 1.09–2.58). The FHG group also showed poorer neurodevelopmental outcomes compared to the HGG, with significant negative associations with DQ in fine motor, cognitive ability, and language ability (aBeta ranging from −2.28 to −3.21). Further, significant associations were observed between FHG and increased risk of DD in gross motor and cognitive abilities (aOR = 2.19, 95% CI: 1.09–4.40; aOR = 2.33, 95% CI: 1.44–3.76, respectively). The unadjusted associations between glycemic patterns and neurodevelopmental outcomes are summarized in Table S7.

### 3.5. Sex-Specific Effects of Glycemic Indicators on Neurodevelopment

**Figure 3:**
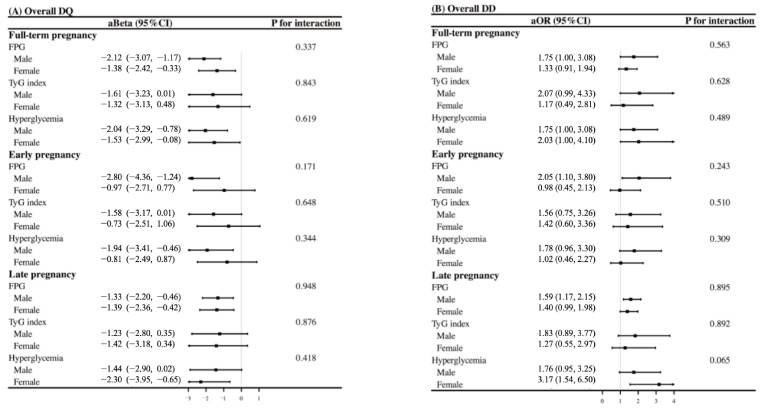
The sex-specific effects of glycemia indicators across early and late pregnancy on overall neurodevelopment. (**A**) illustrates the impact of glycemic indicators on overall developmental quotient (DQ) in male and female offspring. (**B**) presents the effects of glycemic indicators on the risk of overall developmental delay (DD) among boys and girls.

Stratified analysis indicated sex-specific effects of glycemic indicators on neurobehavioral development. In early pregnancy, negative associations between FPG, hyperglycemia, and lower overall DQ were observed only in male offspring (aBeta = −2.80, 95% CI: −4.36 to −1.28; aBeta = −1.94, 95% CI: −3.41 to −0.46, Figure 3A). In late pregnancy, however, hyperglycemia was significantly associated with lower overall DQ only in female offspring (aBeta = −2.30, 95% CI: −3.95 to −0.65), though this association was marginally significant in males (aBeta = −1.08, 95% CI: −2.90 to 0.02). Additionally, significant associations between FPG levels in both early and late pregnancy and the risk of overall developmental delay were observed only in male offspring (aOR = 2.05, 95% CI: 1.10–3.80; aOR = 1.59, 95% CI: 1.17–2.15, Figure 3B). The sex-specific effects of glycemic indicators on specific neurodevelopmental domains are presented in Figures S1–S5.

### 3.6. Sensitivity Analyses for Maternal Age and Folate Intake

Two sensitivity analyses were conducted to ensure the robustness of our results by further controlling for the potential bias of maternal age and folate intake. After excluding pregnant women above 35 years of age, similar findings were obtained. The FPG levels and hyperglycemia both in early and late pregnancy were consistently negatively correlated with offspring DQ in overall development (Tables S8 and S9). In women with regular folate intake, the associations between glycemic indicators and neurobehavioral development of offspring were found to be robust (Tables S10 and S11).

## 4. Discussion

In this study, we found that maternal glycemic indicators in both early and late pregnancy significantly impacted offspring neurodevelopment. Elevated FPG and hyperglycemia were associated with lower overall DQ and a higher risk of DD, particularly affecting fine motor, language, and social behavior domains. The triglyceride–glucose (TyG) index, reflecting insulin resistance, also correlated with reduced DQ and increased DD risk, especially in late pregnancy. Offspring of mothers with hyperglycemia—whether during early or late pregnancy, or persistently—faced greater risks of DD compared to those in the healthy glycemia group. These findings highlight the importance of managing maternal glycemia at multiple pregnancy stages for optimal neurodevelopment in children.

In the present study, we investigated the association between maternal glycemia levels and neurobehavior development in offspring. To our knowledge, this is the first study to distinguish the effects of maternal glucose on neurodevelopment outcomes across multiple pregnancy stages. Several previous studies have indicated that GDM is associated with poorer neurodevelopmental outcomes in offspring [24,25]. Consistent with these findings, our study observed a positive association between FPG levels, hyperglycemia, and DD in offspring. However, a previous study showed that GDM did not increase the risk of neurodevelopmental impairment in offspring at 18 months [26]. Notably, the PREOBE cohort study included only 331 Spanish mother–child pairs. The inconsistencies among these studies could be attributed to differences in sample size and ethnicity. On the other hand, most of the previous studies have primarily focused on the association between FPG during the second or third trimester and DD. In contrast, our study is the first to simultaneously explore the effect of glycemia in multiple stages of pregnancy on neurodevelopment. We found that hyperglycemia in the early pregnancy significantly impacted the development of cognitive ability. Therefore, FPG levels should be considered for pregnant women in the early stages of pregnancy. Even though several studies have reported the risk effects of GDM on DD, some offspring with DD have not been exposed to GDM in utero. Beyond conferring protection to neurodevelopment, the maintenance of glycemic control in the early gestational period serves to diminish the risk of subclinical myocardial dysfunction, not only for women with GDM but also for the fetuses and infants of such mothers [27,28]. Thus, it is necessary to further investigate the association between maternal continuous glucose levels and DD. One study with 1036 mother–child pairs showed that maternal continuous FPG levels at 24–28 gestational weeks were associated with delays in the communication domain [13]. In our study, FPG levels in late pregnancy were negatively associated with language development in offspring. Furthermore, hyperglycemia in the late pregnancy was associated with the risk of the developmental delay in social behavior. Autism is characterized by social avoidance and language barriers [29]. Our findings indicated that offspring of mothers with higher FPG levels in the late pregnancy may need increased attention to their social and language development to mitigate the subsequent risk of autism.

In our study, we categorized maternal glycemic patterns into four distinct groups based on glycemic status across different stages of pregnancy. This classification enabled us to examine how variations in maternal glucose levels throughout pregnancy affect neurodevelopmental outcomes in offspring. Our findings showed that hyperglycemia in late pregnancy was associated with an increased risk of developmental delays. Notably, the full-term hyperglycemia group, characterized by persistent hyperglycemia, exhibited the highest risk, with lower developmental quotients across multiple domains. Interestingly, hyperglycemia occurring only in early pregnancy did not increase the risk of poorer developmental outcomes, highlighting the potential cumulative impact of prolonged elevated glucose exposure on neurodevelopment. These results suggest that managing blood glucose levels in later pregnancy stages may help mitigate the neurodevelopmental risks associated with early pregnancy hyperglycemia. Previous research has primarily focused on the effects of gestational diabetes mellitus during later stages, often overlooking the impact of early pregnancy glycemic levels. Our study highlights that glycemic control in the early stages is equally critical for mitigating risks associated with neurodevelopmental delays. By analyzing glycemic patterns, we provide evidence that continuous monitoring and management of maternal glucose levels at all stages of pregnancy are essential for promoting optimal neurodevelopmental outcomes in children.

Previous studies have demonstrated sex-specific differences in abnormal neurodevelopment. A prospective study in Copenhagen showed that female offspring had better language and cognitive neurodevelopmental scores compared to male offspring in early life. Additionally, males are at a 2–4 times higher risk for neurodevelopmental disorders, such as autism and attention-deficit hyperactivity disorder (ADHD) [30,31,32]. Consistent with these findings, our study showed that elevated FPG levels in early pregnancy were associated with an increased risk of DD specifically in male offspring. The underlying mechanisms for these sex-specific effects on neurodevelopment remain unclear, yet emerging research suggests several potential explanations. One animal study indicated that placental insulin receptors may contribute to cortical developmental delays in male fetuses but not in females, potentially due to physiological and metabolic differences between sexes [33]. Additionally, recent genetic research has proposed the “female protective model,” which suggests that females require a higher threshold of genetic variations, such as copy number variations, to exhibit neurodevelopmental disorders like autism [34,35]. This genetic perspective offers insight into why males are generally more susceptible to these disorders. Hormonal differences during pregnancy may also contribute to the varying sensitivity of male and female fetuses to maternal hyperglycemia [36,37,38]. Metabolic disturbances in early pregnancy can lead to elevated levels of placental hormones such as estrogen, progesterone, and prolactin, as well as increased insulin secretion [39,40]. In contrast, disturbances in mid-to-late pregnancy are associated with elevated levels of placental prolactin and placental growth hormone, along with insulin resistance. These hormonal fluctuations may affect male and female fetuses differently, influencing their susceptibility to neurodevelopmental impacts from maternal hyperglycemia. Together, our findings suggested the importance of considering sex-specific factors in the study of maternal glycemia and neurodevelopment, as males and females may respond differently to both genetic and environmental influences during prenatal development.

This study has several strengths. To our knowledge, it is the first to simultaneously describe the relationship between maternal glycemia indicators across multiple pregnant stages and neurodevelopment in offspring. The China Developmental Scale for Children, an indigenous assessment tool specifically designed for Chinese children, was used, and all offspring were evaluated by professional pediatricians, effectively controlling for misclassification of DD. Additionally, our study utilized a significantly larger sample size than previous studies, allowing us to assess the effects of glycemic pattern on the neurobehavioral development in offspring. Furthermore, this is the first study to comprehensively evaluate the effect of the TyG index, an important biomarker of insulin resistance, on neurodevelopmental outcomes in offspring. Last, our study investigated the sex-specific effects of glycemia indicators across early and late pregnancy on the neurodevelopment in offspring, providing new insights into how maternal glucose levels may differentially impact male and female offspring.

This study has several limitations that must be taken into consideration. First, the confounding effects of smoking and drinking status were not controlled due to the high missing rate of these two covariables. However, previous studies indicate that the prevalence of smoking and drinking is less than 3.5% among women in Tongzhou, suggesting that the impact of these potential confounders is likely minimal. Second, dietary behavior data were not collected in this study, which may have influenced maternal glycemic levels and, consequently, the outcomes in offspring. Future studies should incorporate detailed dietary assessments to better understand the role of maternal nutrition in neurodevelopmental outcomes. Third, data on glycemic disorder treatment during pregnancy and its potential impact on fasting plasma glucose levels and offspring outcomes were not collected. Fourth, all mother–child pairs were from Beijing in our single-site study. As such, the findings may not be generalizable to other countries or regions with different maternal metabolic profiles or healthcare systems. It is necessary to investigate the association between maternal metabolism and neurodevelopmental delays in other regions of China, particularly in less developed central and western areas. Fifth, hyperglycemia in this study was defined using a relatively low threshold (FPG > 5.1 mmol/L), which is stricter than thresholds used in some other studies. Results may differ with higher glucose values, and further research using varying definitions of hyperglycemia is warranted. Finally, while our birth cohort study revealed some novel associations between maternal metabolism and neurodevelopment in offspring, the biological mechanisms underlying these associations need to be urgently explored in future studies.

## 5. Conclusions

This study highlights the significant impact of maternal glycemia in both early and late pregnancy on offspring neurodevelopment. Elevated FPG and hyperglycemia were linked to lower DQ and an increased risk of DD across various domains. The TyG index also correlated with reduced DQ and higher DD risk. As the first study to assess glycemic effects across multiple pregnancy stages, it emphasizes the importance of effective glycemic management throughout pregnancy for optimizing neurodevelopmental outcomes. Notably, maternal hyperglycemia had a greater impact on male offspring. These findings underscore the need for continuous blood glucose monitoring and individualized care to improve long-term neurodevelopmental outcomes.

## Figures and Tables

**Table 1:** Characteristics of mothers and offspring by neurodevelopment status.

Characteristic	Overall	ND	DD	
	N = 1888	N = 1791	N = 97	*p*
Maternal age, mean (SD), years	28.2 (3.7)	28.2 (3.7)	28.8 (4.0)	0.2
Educational levels (%)				0.014
≤2 years	380 (20.1)	351 (19.6)	29 (29.9)	
>12 years	1508 (79.9)	1440 (80.4)	68 (70.1)	
Occupation (%)				0.029
Blue collar	476 (25.2)	460 (25.7)	16 (16.5)	
White collar	921 (48.8)	875 (48.9)	46 (47.4)	
Freelancer	491 (26.0)	456 (25.5)	35 (36.1)	
Parity (%)				0.001
Primiparity	1602 (84.9)	1531 (85.5)	71 (73.2)	
Multiparity	286 (15.1)	260 (14.5)	26 (26.8)	
Pre-pregnant BMI (%)				0.021
≤25	1580 (83.7)	1507 (84.1)	73 (75.3)	
>25	308 (16.3)	284 (15.9)	24 (24.7)	
Folate intake (%)				0.2
Regular intake	1767 (93.6)	1679 (93.7)	88 (90.7)	
Irregular intake	121 (6.4)	112 (6.3)	9 (9.3)	
Season of conception (%)				0.4
Spring	405 (21.5)	380 (21.2)	25 (25.8)	
Summer	587 (31.1)	560 (31.3)	27 (27.8)	
Autumn	478 (25.3)	450 (25.1)	28 (28.9)	
Winter	418 (22.1)	401 (22.4)	17 (17.5)	
Sex of offspring (%)				0.2
Male	1039 (55.0)	979 (54.7)	60 (61.9)	
Female	849 (45.0)	812 (45.3)	37 (38.1)	
Gestational age at birth (SD), weeks	39.1 (2.0)	39.2 (1.8)	37.6 (3.2)	<0.001
Birthweight, mean (SD), grams	3310.0 (542.5)	3328.9 (517.7)	2961.3 (811.8)	<0.001

**Table 2:** Full-term glycemic indicators and neurobehavioral outcomes.

Neurodevelopment	Glycemia Indicator	DQ			DD		
		aBeta	95% CI	*p*	aOR	95% CI	*p*
Overall development	FPG (mmol/L)	−0.97	(−1.67, −0.28)	0.006	1.37	(1.07, 1.76)	0.012
	TyG index	−1.53	(−2.70, −0.36)	0.010	1.69	(0.97, 2.93)	0.064
	Glycemic status						
	Normoglycemic	ref	ref	ref	ref	ref	ref
	Hyperglycemic	−1.13	(−2.05, −0.21)	0.017	1.68	(1.07, 2.64)	0.022
Gross motor	FPG (mmol/L)	−0.32	(−1.14, 0.49)	0.434	1.17	(0.88, 1.56)	0.272
	TyG index	−0.89	(−2.26, 0.48)	0.203	1.53	(0.86, 2.73)	0.147
	Glycemic status						
	Normoglycemic	ref	ref	ref	ref	ref	ref
	Hyperglycemic	−0.65	(−1.74, 0.43)	0.239	1.45	(0.91, 2.31)	0.117
Fine motor	FPG (mmol/L)	−0.92	(−1.71, −0.14)	0.021	1.31	(1.05, 1.63)	0.015
	TyG index	−1.30	(−2.63, 0.02)	0.054	1.48	(0.95, 2.29)	0.083
	Glycemic status						
	Normoglycemic	ref	ref	ref	ref	ref	ref
	Hyperglycemic	−0.68	(−1.73, 0.37)	0.207	1.36	(0.95, 1.93)	0.092
Cognitive ability	FPG (mmol/L)	−1.06	(−2.15, 0.03)	0.056	1.16	(0.94, 1.43)	0.162
	TyG index	−1.29	(−3.12, 0.55)	0.170	1.02	(0.67, 1.54)	0.930
	Glycemic status						
	Normoglycemic	ref	ref	ref	ref	ref	ref
	Hyperglycemic	−1.12	(−2.58, 0.33)	0.130	1.35	(0.98, 1.86)	0.069
Language ability	FPG (mmol/L)	−1.17	(−2.12, −0.23)	0.015	1.24	(0.99, 1.57)	0.066
	TyG index	−1.62	(−3.21, −0.03)	0.045	1.40	(0.90, 2.24)	0.132
	Glycemic status						
	Normoglycemic	ref	ref	ref	ref	ref	ref
	Hyperglycemic	−1.45	(−2.71, −0.19)	0.024	1.18	(0.82, 1.71)	0.367
Social behavior	FPG (mmol/L)	−1.38	(−2.48, −0.27)	0.015	1.28	(1.04, 1.59)	0.022
	TyG index	−2.55	(−4.41, −0.68)	0.007	1.24	(0.81, 1.91)	0.319
	Glycemic status						
	Normoglycemic	ref	ref	ref	ref	ref	ref
	Hyperglycemic	−1.74	(−3.22, −0.26)	0.021	1.23	(0.87, 1.73)	0.237

DQ: neurodevelopment quotient; DD: neurodevelopment delay; aBeta: adjusted Beta; aOR: adjusted OR; FPG: fasting plasma glucose; TyG: triglyceride glucose; ref: reference group; hyperglycemia was defined as the FPG > 5.1 mmol/L. aBeta and aOR were adjusted by the covariables including maternal age, educational level, occupation, parity, pre-pregnancy BMI, folate intake, season of conception, sex of the offspring, gestational age, birthweight, and age of the offspring.

**Table 3:** Associations between patterns of glycemic changes during pregnancy and neurobehavioral development in offspring.

Neurodevelopment	Glycemia Group	DQ			DD		
		aBeta	95% CI	*p*	aOR	95% CI	*p*
Overall development	HGG	ref	ref	ref	ref	ref	ref
	EHG	−1.35	(−2.70, 0.01)	0.051	1.18	(0.6, 2.32)	0.622
	LHG	−1.84	(−3.13, −0.54)	0.006	2.18	(1.26, 3.77)	0.005
	FHG	−2.40	(−4.12, −0.67)	0.006	2.64	(1.38, 5.05)	0.003
Gross motor	HGG	ref	ref	ref	ref	ref	ref
	EHG	−1.28	(−2.84, 0.27)	0.106	1.55	(0.83, 2.9)	0.174
	LHG	−0.42	(−1.91, 1.07)	0.577	1.35	(0.71, 2.58)	0.363
	FHG	−1.81	(−3.78, 0.17)	0.074	2.19	(1.09, 4.4)	0.028
Fine motor	HGG	ref	ref	ref	ref	ref	ref
	EHG	−0.41	(−1.93, 1.12)	0.602	1.28	(0.78, 2.1)	0.337
	LHG	−1.62	(−3.08, −0.16)	0.030	1.62	(1.03, 2.54)	0.036
	FHG	−2.28	(−4.22, −0.34)	0.021	1.78	(1.01, 3.13)	0.046
Cognitive ability	HGG	ref	ref	ref	ref	ref	ref
	EHG	−2.09	(−4.19, 0.01)	0.051	1.31	(0.83, 2.08)	0.249
	LHG	−1.09	(−3.10, 0.92)	0.288	1.3	(0.84, 2.01)	0.239
	FHG	−3.21	(−5.88, −0.53)	0.019	2.33	(1.44, 3.76)	<0.001
Language ability	HGG	ref	ref	ref	ref	ref	ref
	EHG	−1.46	(−3.27, 0.35)	0.113	0.95	(0.55, 1.65)	0.863
	LHG	−2.16	(−3.89, −0.44)	0.014	1.56	(0.98, 2.48)	0.061
	FHG	−2.57	(−4.86, −0.27)	0.029	1.37	(0.73, 2.57)	0.324
Social behavior	HGG	ref	ref	ref	ref	ref	ref
	EHG	−1.50	(−3.66, 0.65)	0.172	0.86	(0.51, 1.47)	0.587
	LHG	−3.88	(−5.95, −1.82)	<0.001	1.68	(1.09, 2.58)	0.018
	FHG	−2.13	(−4.87, 0.61)	0.128	1.67	(0.95, 2.92)	0.073

DQ: neurodevelopment quotient; DD: neurodevelopment delay; aBeta: adjusted Beta; aOR: adjusted OR; HGG: healthy glycemia group; EHG: early pregnancy hyperglycemia group; LHG: late pregnancy hyperglycemia group, FHG: full-term hyperglycemia group. aBeta and aOR were adjusted by the covariables including maternal age, educational level, occupation, parity, pre-pregnancy BMI, folate intake, season of conception, sex of the offspring, gestational age, birthweight, and age of the offspring.

## Data Availability

The data that support the findings of this study are not openly available due to reasons of sensitivity and are available from the corresponding author upon reasonable request. Data are located in controlled access data storage at Peking University.

## Supplementary Materials

The following supporting information can be downloaded at: https://www.mdpi.com/article/10.3390/nu17020257/s1, Figure S1: The forestplot for the sex-specific effects of glycemia indicators on the development of gross motor; Figure S2. The forestplot for the sex-specific effects of glycemia indicators on the development of fine motor; Figure S3. The forestplot for the sex-specific effects of glycemia indicators on the development of cognitive ability; Figure S4. The forestplot for the sex-specific effects of glycemia indicators on the development of language ability; Figure S5. The forestplot for the sex-specific effects of glycemia indicators on the development of social behavior; Table S1. Characteristics of mothers and offspring by hyperglycemia status; Table S2. The characteristic glycemia condition in different stage of pregnancy; Table S3. The characteristic of Development quotient in the offspring; Table S4. The unadjusted relationships between glycemic indicators in full-term pregnancy and neurodevelopmental outcomes; Table S5. The unadjusted relationships between glycemic indicators in early pregnancy and neurodevelopmental outcomes; Table S6. The unadjusted relationships between glycemic indicators in late pregnancy and neurodevelopmental outcomes; Table S7. The unadjusted associations between glycemic patterns and neurodevelopmental outcomes; Table S8. The associations between glycemia indicators in women under 35 years old and neurodevelopment quotient; Table S9. The associations between glycemia indicators in women under 35 years old and the risk of DD; Table S10. The associations between glycemia indicators in women with regular folate intake and neurodevelopment quotient; Table S11. The associations between glycemia indicators in women with regular filate intake and the risk of DD.
