# Supplementary material for: Maternal Glycemia and Its Pattern Associated with Offspring Neurobehavioral Development: A Chinese Birth Cohort Study
Source: Nutrients. 2025 Jan 11;17(2):257. doi: 10.3390/nu17020257 (PMC11767945; doi:10.3390/nu17020257)
Supplement: Supplementary file 1 [file nutrients-17-00257-s001.zip › nutrients-3373529-supplementary.pdf]

Supplementary Material

Title: Maternal Glycemia and Its Pattern Associated with Offspring's Neurobehavioral Development: A Chinese Birth Cohort Study

Zhichao Yuan[1], Tao Su[2], Li Yang[2], Lei Xi[2], Hai-Jun Wang[1], Yuelong Ji[1]

[1]Department of Maternal and Child Health, School of Public Health, Peking University, Beijing, 100191, China.

[2]Tongzhou Maternal and Child Health Care Hospital of Beijing, Beijing, 101101, China.

*Corresponding author:

Yuelong Ji, PhD, MPH, MSc

Department of Maternal and Child Health, School of Public Health, Peking University

38 Xueyuan Road, Haidian District, Beijing

Phone: +86 13161989008

mail: yuelong.ji@pku.edu.cn

This file includes:

Table S1. Characteristics of mothers and offspring by hyperglycemia status

Table S2. The characteristic glycemia condition in different stage of pregnancy

Table S3. The characteristic of Development quotient in the offspring

Table S4. The unadjusted relationships between glycemic indicators in full-term pregnancy and neurodevelopmental outcomes

Table S5. The unadjusted relationships between glycemic indicators in early pregnancy and neurodevelopmental outcomes

Table S6. The unadjusted relationships between glycemic indicators in late pregnancy and neurodevelopmental outcomes

Table S7. The unadjusted associations between glycemic patterns and neurodevelopmental outcomes

Table S8. The associations between glycemia indicators in women under 35 years old and neurodevelopment quotient

Table S9. The associations between glycemia indicators in women under 35 years old

and the risk of DD

Table S10. The associations between glycemia indicators in women with regular folate intake and neurodevelopment quotient

Table S11. The associations between glycemia indicators in women with regular filate intake and the risk of DD

Figure S1. The forestplot for the sex-specific effects of glycemia indicators on the development of gross motor

Figure S2. The forestplot for the sex-specific effects of glycemia indicators on the development of fine motor

Figure S3. The forestplot for the sex-specific effects of glycemia indicators on the development of cognitive ability

Figure S4. The forestplot for the sex-specific effects of glycemia indicators on the development of language ability

Figure S5. The forestplot for the sex-specific effects of glycemia indicators on the development of social behavior

sTable 1. Characteristics of mothers and offspring by hyprclycemia status

Characteristic	Early pregnacy			Late pregnancy		
	Normalglycemic	Hyperglycemic		Normalglycemic	Hyperglycemic	
	N=1,505	N=383	P	N=1,791	N=97	P
Maternal age, mean (SD), years	28.1 (3.7)	28.8 (3.7)	0.003	28.0 (3.5)	28.9 (4.1)	<0.001
Educational levels (%)			0.200			0.300
<= 12 years	311 (20.7)	69 (18.0)		287 (19.6)	93 (22.0)	
> 12 years	1,194 (79.3)	68 (70.1)		1,178 (80.4)	330 (78.0)	
Occupation (%)			0.037			0.100
Blue collar	394 (26.2)	82 (21.4)		385 (26.3)	91 (21.5)	
White collar	737 (49.0)	184 (48.0)		710 (48.5)	211 (49.9)	
Freelancer	374 (24.9)	117 (30.5)		370 (25.3)	121 (28.6)	
Parity (%)			0.400			0.001
Primiparity	1,282 (85.2)	320 (83.6)		1,250 (85.3)	352 (83.2)	
Multiparity	223 (14.8)	63 (16.4)		215 (14.7)	71 (16.8)	
Pre-pregnant BMI(%)			<0.001			<0.001
<=25	1,291 (85.8)	289 (75.5)		1,279 (87.3)	301 (71.2)	
>25	214 (14.2)	94 (24.5)		186 (12.7)	122 (28.8)	
Folate intake (%)			0.130			0.400
Regular intake	1,402 (93.2)	365 (95.3)		1,375 (93.9)	392 (92.7)	
Irregular intake	103 (6.8)	18 (4.7)		90 (6.1)	31 (7.3)	
Season of conception (%)			0.012			0.024
Spring	335 (22.3)	70 (18.3)		299 (20.4)	106 (25.1)	
Summer	484 (32.2)	103 (26.9)		447 (30.5)	140 (33.1)	
Autumn	362 (24.1)	116 (30.3)		392 (26.8)	86 (20.3)	
Winter	324 (21.5)	94 (24.5)		327 (22.3)	91 (21.5)	

Sex of offspring (%)			0.700			0.700
Male	825 (54.8)	214 (55.9)		810 (55.3)	229 (54.1)	
Female	680 (45.2)	169 (44.1)		655 (44.7)	194 (45.9)	
Gestational age at birth (SD), weeks	39.1 (1.9)	38.9 (2.2)	0.047	39.3 (1.8)	38.4 (2.4)	<0.001
Birthweight, mean (SD), grams	3,313.5 (524.2)	3,296.3 (609.7)	>0.900	3,341.6 (502.4)	3,200.8 (651.9)	0.004

sTable 2. The characteristic glycemia condition in different stage of pregnancy

Pregnant stage	Glycemia indicators	Overall	ND	DD	
		N=1,888	N=1,791	N=97	P
Full-term pregnancy	FPG (mmol/L)	5.08 (0.66)	5.07 (0.62)	5.4 (1.09)	0.002
	Tyg index	5.01 (0.38)	5.01 (0.37)	5.09 (0.49)	0.200
	Glycemic status				<0.001
	Normalglycemic	1209 (64.04)	1163 (64.94)	46 (47.42)	
	Hyperglycemic	679 (35.96)	628 (35.06)	51 (52.58)	
Early pregnancy	FPG (mmol/L)	4.84 (0.4)	4.84 (0.39)	4.94 (0.52)	0.074
	Tyg index	3.79 (0.4)	3.79 (0.4)	3.9 (0.49)	0.054
	Glycemic status				0.055
	Normalglycemic	1489 (78.87)	1420 (79.29)	69 (71.13)	
	Hyperglycemic	399 (21.13)	371 (20.71)	28 (28.87)	
Late pregnancy	FPG (mmol/L)	4.86 (0.71)	4.84 (0.68)	5.17 (1.17)	0.036
	Tyg index	4.97 (0.39)	4.96 (0.38)	5.05 (0.51)	0.200
	Glycemic status				<0.001
	Normalglycemic	1458 (77.22)	1400 (78.17)	58 (59.79)	
	Hyperglycemic	430 (22.78)	391 (21.83)	39 (40.21)	

ND: normal development; DD: neurodevelopmental delay; early pregnancy: <13 gestational week; Late pregnancy：≥13 gestational week; FPG: fasting plasma glucose; TyG: triglyceride glucose; Hyperglycemia was defined as the FPG >5.1 mmol/L.

sTable 3. The characteristic of Development quotient in the offspring

Development quotient mean (SD)	All offspring	Female offspring	Male offspring	P
Overall development	97.72 (10.5)	98.52 (10.21)	97.06 (10.7)	0.006
Gross motor	97.35 (11.42)	97.71 (11.55)	97.07 (11.31)	0.200
Fine motor	94.88 (11.67)	96.04 (10.4)	93.93 (12.54)	<0.001
Cognitive ability	97.86 (15.41)	98.77 (14.86)	97.13 (15.82)	0.047
Language ability	97.15 (14.03)	98.3 (14.32)	96.21 (13.72)	<0.001
Social behavior	101.34 (17.71)	101.79 (18.03)	100.97 (17.45)	0.300

sTable 4. The unadjusted relationships between glycemic indicators in full-term pregnancy and neurodevelopmental outcomes

Neurodevelopment	Glycemia indicator	DQ			DD		
		cBeta	95% CI	P	cOR	95% CI	P
Overall development	FPG (mmol/L)	-1.98	(-2.70, -1.27)	<0.001	1.63	(1.31, 2.02)	<0.001
	Tyg index	-2.11	(-3.36, -0.86)	<0.001	1.76	(1.05, 2.95)	0.031
	Glycemic status						
	Normalglycemic	ref	ref	ref	ref	ref	ref
	Hyperglycemic	-2.07	(-3.06, -1.09)	<0.001	2.05	(1.36, 3.10)	<0.001
Gross motor	FPG (mmol/L)	-1.10	(-1.88, -0.32)	0.006	1.38	(1.07, 1.77)	0.013
	Tyg index	-1.10	(-2.45, 0.27)	0.116	1.62	(0.92, 2.84)	0.095
	Glycemic status						
	Normalglycemic	ref	ref	ref	ref	ref	ref
	Hyperglycemic	-1.09	(-2.38, -0.24)	0.017	1.70	(1.09, 2.65)	0.020
Fine motor	FPG (mmol/L)	-1.95	(-2.25, -1.15)	<0.001	1.54	(1.28, 1.85)	<0.001
	Tyg index	-1.97	(-3.36, -0.58)	0.005	1.68	(1.12, 2.53)	0.012
	Glycemic status						
	Normalglycemic	ref	ref	ref	ref	ref	ref
	Hyperglycemic	-1.71	(-2.81, -0.62)	0.002	1.65	(1.20, 2.28)	0.002
Cognitive ability	FPG (mmol/L)	-2.25	(-3.31, -1.20)	<0.001	1.41	(1.17, 1.70)	<0.001
	Tyg index	-1.75	(-3.58, 0.09)	0.062	1.17	(0.78, 1.73)	0.450
	Glycemic status						
	Normalglycemic	ref	ref	ref	ref	ref	ref
	Hyperglycemic	-2.24	(-3.68, -0.79)	0.002	1.65	(1.21, 2.24)	0.001
Language ability	FPG (mmol/L)	-2.03	(-2.99, -1.07)	<0.001	1.38	(1.14, 1.68)	0.001
	Tyg index	-2.32	(-3.99, -0.65)	0.007	1.59	(1.05, 2.41)	0.028
	Glycemic status						
	Normalglycemic	ref	ref	ref	ref	ref	ref
	Hyperglycemic	-2.27	(-3.59, -0.95)	<0.001	1.37	(0.99, 1.90)	0.061
Social behavior	FPG (mmol/L)	-2.59	(-3.81, -1.38)	<0.001	1.42	(1.18, 1.71)	<0.001
	Tyg index	-3.44	(-5.54, -1.33)	0.001	1.37	(0.93, 2.04)	0.114
	Glycemic status						
	Normalglycemic	ref	ref	ref	ref	ref	ref
	Hyperglycemic	-2.84	(-4.50, -1.18)	<0.001	1.42	(1.04, 1.93)	0.028

DQ: neurodevelopment quotient; DD: neurodevelopment delay; cBeta: crude Beta; cOR: crude OR; FPG: fasting plasma glucose; TyG: triglyceride glucose; Hyperglycemia was defined as the FPG >5.1 mmol/L

sTable 5. The unadjusted relationships between glycemic indicators in early pregnancy and neurodevelopmental outcomes

Neurodevelopment	Glycemia indicator	DQ			DD		
		cBeta	95% CI	P	cOR	95% CI	P
Overall development	FPG (mmol/L)	-2.34	(-3.53, -1.15)	<0.001	1.70	(1.10, 2.63)	0.016
	Tyg index	-2.03	(-3.20, -0.85)	<0.001	1.91	(1.19, 3.09)	0.008
	Glycemic status						
	Normalglycemic	ref	ref	ref	ref	ref	ref
	Hyperglycemic	-1.61	(-2.77, -0.45)	0.006	1.55	(0.99, 2.44)	0.058
Gross motor	FPG (mmol/L)	-2.05	(-3.35, -0.76)	0.002	1.58	(0.98, 2.56)	0.061
	Tyg index	-1.22	(-2.49, 0.06)	0.062	1.24	(0.72, 2.13)	0.437
	Glycemic status						
	Normalglycemic	ref	ref	ref	ref	ref	ref
	Hyperglycemic	-1.67	(-2.93, -0.41)	0.009	1.82	(1.12, 2.94)	0.015
Fine motor	FPG (mmol/L)	-1.72	(-3.05, -0.40)	0.011	1.57	(1.10, 2.24)	0.014
	Tyg index	-1.87	(-3.18, -0.57)	0.005	1.88	(1.28, 2.74)	0.001
	Glycemic status						
	Normalglycemic	ref	ref	ref	ref	ref	ref
	Hyperglycemic	-1.14	(-2.43, 0.15)	0.083	1.44	(1.00, 2.06)	0.049
Cognitive ability	FPG (mmol/L)	-3.16	(-4.9, -1.41)	<0.001	1.60	(1.14, 2.26)	0.007
	Tyg index	-2.07	(-3.8, -0.35)	0.019	1.42	(0.98, 2.05)	0.063
	Glycemic status						
	Normalglycemic	ref	ref	ref	ref	ref	ref
	Hyperglycemic	-2.42	(-4.12, -0.72)	0.005	1.68	(1.20, 2.36)	0.002
Language ability	FPG (mmol/L)	-2.42	(-4.01, -0.82)	0.003	1.29	(0.88, 1.89)	0.197
	Tyg index	-2.22	(-3.79, -0.66)	0.006	1.59	(1.08, 2.35)	0.019
	Glycemic status						
	Normalglycemic	ref	ref	ref	ref	ref	ref
	Hyperglycemic	-1.63	(-3.18, -0.08)	0.039	1.10	(0.75, 1.62)	0.629
Social behavior	FPG (mmol/L)	-2.37	(-4.38, -0.36)	0.021	1.46	(1.03, 2.08)	0.033
	Tyg index	-2.74	(-4.72, -0.76)	0.007	1.38	(0.95, 2.00)	0.087
	Glycemic status						
	Normalglycemic	ref	ref	ref	ref	ref	ref
	Hyperglycemic	-1.19	(-3.15, 0.76)	0.232	1.09	(0.76, 1.58)	0.631

DQ: neurodevelopment quotient; DD: neurodevelopment delay; cBeta: crude Beta; cOR: crude OR; FPG: fasting plasma glucose; TyG: triglyceride glucose; Hyperglycemia was defined as the FPG >5.1 mmol/L

sTable 6. The unadjusted relationships between glycemic indicators in late pregnancy and neurodevelopmental outcomes

Neurodevelopment	Glycemia indicator	DQ			DD		
		cBeta	95% CI	P	cOR	95% CI	P
Overall development	FPG (mmol/L)	-1.93	(-3.14, -0.71)	<0.001	1.54	(1.26, 1.88)	<0.001
	Tyg index	-2.10	(-3.22, -0.97)	0.002	1.71	(1.04, 2.82)	0.036
	Glycemic status						
	Normalglycemic	ref	ref	ref	ref	ref	ref
	Hyperglycemic	-0.62	(-1.26, 0.02)	<0.001	2.41	(1.58, 3.68)	<0.001
Gross motor	FPG (mmol/L)	-0.73	(-1.45, -0.01)	0.048	1.33	(1.05, 1.69)	0.020
	Tyg index	-0.90	(-2.22, 0.42)	0.181	1.58	(0.91, 2.73)	0.104
	Glycemic status						
	Normalglycemic	ref	ref	ref	ref	ref	ref
	Hyperglycemic	-0.96	(-2.18, 0.27)	0.127	1.55	(0.95, 2.51)	0.076
Fine motor	FPG (mmol/L)	-1.52	(-2.26, -0.79)	<0.001	1.40	(1.17, 1.67)	<0.001
	Tyg index	-1.79	(-3.14, -0.44)	0.009	1.56	(1.05, 2.32)	0.027
	Glycemic status						
	Normalglycemic	ref	ref	ref	ref	ref	ref
	Hyperglycemic	-2.14	(-3.39, -0.89)	<0.001	1.73	(1.23, 2.44)	0.002
Cognitive ability	FPG (mmol/L)	-1.66	(-2.63, -0.69)	<0.001	1.35	(1.14, 1.61)	<0.001
	Tyg index	-1.51	(-3.29, 0.28)	0.099	1.16	(0.79, 1.71)	0.454
	Glycemic status						
	Normalglycemic	ref	ref	ref	ref	ref	ref
	Hyperglycemic	-1.98	(-3.64, -0.33)	0.019	1.75	(1.26, 2.43)	<0.001
Language ability	FPG (mmol/L)	-1.64	(-2.52, -0.75)	<0.001	1.36	(1.13, 1.63)	<0.001
	Tyg index	-2.17	(-3.79, -0.54)	0.009	1.59	(1.06, 2.39)	0.024
	Glycemic status						
	Normalglycemic	ref	ref	ref	ref	ref	ref
	Hyperglycemic	-2.33	(-3.84, -0.82)	0.002	1.58	(1.11, 2.25)	0.012
Social behavior	FPG (mmol/L)	-3.07	(-4.97, -1.16)	<0.001	1.34	(1.13, 1.60)	<0.001
	Tyg index	-3.26	(-5.31, -1.21)	0.002	1.34	(0.91, 1.97)	0.133
	Glycemic status						
	Normalglycemic	ref	ref	ref	ref	ref	ref
	Hyperglycemic	-3.07	(-4.97, -1.16)	0.002	1.73	(1.24, 2.41)	0.001

DQ: neurodevelopment quotient; DD: neurodevelopment delay; cBeta: crude Beta; cOR: crude OR; FPG: fasting plasma glucose; TyG: triglyceride glucose; Hyperglycemia was defined as the FPG >5.1 mmol/L

sTable 7. The unadjusted associations between glycemic patterns and neurodevelopmental outcomes

Neurodevelopment	Glycemia group	DQ			DD		
		cBeta	95% CI	P	cOR	95% CI	P
Overall development	HGG	ref	ref	ref	ref	ref	ref
	EHG	-1.57	(-3.00, -0.15)	0.031	1.28	(0.67, 2.45)	0.457
	LHG	-2.16	(-3.52, -0.80)	0.002	2.26	(1.35, 3.8)	0.002
	FHG	-2.75	(-4.52, -0.97)	0.002	3.02	(1.66, 5.48)	<0.001
Gross motor	HGG	ref	ref	ref	ref	ref	ref
	EHG	-1.48	(-3.04, 0.07)	0.062	1.66	(0.89, 3.08)	0.112
	LHG	-0.53	(-2.01, 0.95)	0.482	1.35	(0.72, 2.56)	0.352
	FHG	-2.29	(-4.23, -0.36)	0.021	2.42	(1.24, 4.70)	0.009
Fine motor	HGG	ref	ref	ref	ref	ref	ref
	EHG	-0.76	(-2.34, 0.83)	0.351	1.36	(0.85, 2.18)	0.204
	LHG	-2.01	(-3.53, -0.50)	0.009	1.7	(1.11, 2.60)	0.014
	FHG	-2.78	(-4.75, -0.80)	0.006	2.07	(1.25, 3.45)	0.005
Cognitive ability	HGG	ref	ref	ref	ref	ref	ref
	EHG	-2.06	(-4.16, 0.04)	0.054	1.32	(0.84, 2.08)	0.230
	LHG	-1.58	(-3.58, 0.42)	0.121	1.41	(0.92, 2.16)	0.113
	FHG	-3.79	(-6.4, -1.18)	0.004	2.72	(1.72, 4.28)	<0.001
Language ability	HGG	ref	ref	ref	ref	ref	ref
	EHG	-1.67	(-3.58, 0.23)	0.086	1.01	(0.61, 1.70)	0.955
	LHG	-2.53	(-4.35, -0.71)	0.006	1.59	(1.04, 2.43)	0.034
	FHG	-2.82	(-5.19, -0.44)	0.020	1.57	(0.91, 2.72)	0.107
Social behavior	HGG	ref	ref	ref	ref	ref	ref
	EHG	-1.9	(-4.31, 0.51)	0.123	0.94	(0.57, 1.55)	0.801
	LHG	-4.13	(-6.43, -1.84)	<0.001	1.69	(1.13, 2.51)	0.010
	FHG	-2.07	(-5.07, 0.93)	0.176	1.75	(1.06, 2.89)	0.030

DQ: neurodevelopment quotient; DD: neurodevelopment delay; cBeta: crude Beta; HGG: healthy glycemia group; EHG: early-pregnancy hyperglycemia group; LHG: late-pregnancy hyperglycemia group, FHG: full-term hyperglycemia group

sTable 8. The associations between glycemia indicators in women under 35 years old and neurodevelopment quotient

Neurodevelopment	Glycemia indicator	Full-term pregnancy			Early pregnacy			Late pregnancy		
		aBeta	95% CI	P	aBeta	95% CI	P	aBeta	95% CI	P
Overall development	FPG (mmol/L)	-1.39	(-2.15, -0.62)	<0.001	-1.74	(-2.92, -0.56)	0.004	-1.07	(-1.77, -0.37)	0.003
	Tyg index	-1.24	(-2.48, 0.00)	0.050	-0.99	(-2.22, 0.23)	0.112	-1.13	(-2.34, 0.08)	0.067
	Glycemic status									
	Normalglycemic	ref	ref	ref	ref	ref	ref	ref	ref	ref
	Hyperglycemic	-1.51	(-2.48, -0.54)	0.002	-1.16	(-2.31, -0.01)	0.047	-1.73	(-2.86, -0.6)	0.003
Gross motor	FPG (mmol/L)	-0.76	(-1.64, 0.13)	0.095	-1.60	(-2.97, -0.23)	0.022	-0.50	(-1.31, 0.32)	0.231
	Tyg index	-1.02	(-2.46, 0.42)	0.165	-0.77	(-2.19, 0.65)	0.288	-0.90	(-2.3, 0.51)	0.211
	Glycemic status									
	Normalglycemic	ref	ref	ref	ref	ref	ref	ref	ref	ref
	Hyperglycemic	-0.89	(-2.02, 0.24)	0.122	-1.19	(-2.52, 0.14)	0.081	-0.82	(-2.13, 0.50)	0.224
Fine motor	FPG (mmol/L)	-1.32	(-2.18, -0.46)	0.003	-1.03	(-2.37, 0.3)	0.130	-1.07	(-1.86, -0.28)	0.008
	Tyg index	-0.99	(-2.39, 0.42)	0.170	-0.64	(-2.02, 0.75)	0.367	-0.92	(-2.29, 0.46)	0.191
	Glycemic status									
	Normalglycemic	ref	ref	ref	ref	ref	ref	ref	ref	ref
	Hyperglycemic	-1.13	(-2.23, -0.03)	0.043	-0.58	(-1.88, 0.71)	0.378	-1.90	(-3.18, -0.62)	0.004
Cognitive ability	FPG (mmol/L)	-1.54	(-2.73, -0.36)	0.011	-2.54	(-4.37, -0.71)	0.007	-1.00	(-2.09, 0.09)	0.072
	Tyg index	-0.96	(-2.89, 0.97)	0.332	-0.91	(-2.81, 0.99)	0.348	-0.74	(-2.62, 1.14)	0.439
	Glycemic status									
	Normalglycemic	ref	ref	ref	ref	ref	ref	ref	ref	ref
	Hyperglycemic	-1.57	(-3.08, -0.06)	0.041	-1.94	(-3.72, -0.16)	0.033	-1.27	(-3.02, 0.49)	0.159
Language ability	FPG (mmol/L)	-1.49	(-2.52, -0.46)	0.004	-1.83	(-3.41, -0.24)	0.024	-1.17	(-2.11, -0.23)	0.015
	Tyg index	-1.33	(-3.00, 0.34)	0.118	-1.07	(-2.72, 0.57)	0.202	-1.23	(-2.86, 0.39)	0.138
	Glycemic status									

	Normalglycemic	ref	ref	ref	ref	ref	ref	ref	ref	ref
	Hyperglycemic	-1.75	(-3.05, -0.44)	0.009	-1.36	(-2.9, 0.19)	0.085	-1.89	(-3.41, -0.37)	0.015
Social behavior	FPG (mmol/L)	-1.83	(-3.05, -0.61)	0.003	-1.69	(-3.58, 0.19)	0.079	-1.59	(-2.71, -0.47)	0.005
	Tyg index	-1.91	(-3.90, 0.08)	0.059	-1.58	(-3.54, 0.38)	0.114	-1.86	(-3.80, 0.07)	0.059
	Glycemic status									
	Normalglycemic	ref	ref	ref	ref	ref	ref	ref	ref	ref
	Hyperglycemic	-2.20	(-3.75, -0.64)	0.006	-0.73	(-2.56, 1.11)	0.436	-2.76	(-4.57, -0.95)	0.003

FPG: fasting plasma glucose; TyG: triglyceride glucose; Hyperglycemia was defined as the FPG >5.1 mmol/L; aBeta was adjusted by the covariables including maternal age, educational level, occupation, parity, pre-pregnancy BMI, folate intake, season of conception, sex of the offspring, gestational age, birthweight, and age of the offspring.

sTable 9. The associations between glycemia indicators in women under 35 years old and the risk of DD

Neurodevelopment	Glycemia indicator	Full-term pregnancy			Early pregnancy			Late pregnancy		
		aOR	95% CI	P	aOR	95% CI	P	aOR	95% CI	P
Overall development	FPG (mmol/L)	1.52	(1.15, 2.01)	0.003	1.43	(0.88, 2.33)	0.150	1.45	(1.11, 1.89)	0.006
	Tyg index	1.40	(0.78, 2.51)	0.262	1.43	(0.81, 2.55)	0.222	1.38	(0.78, 2.43)	0.271
	Glycemic status									
	Normalglycemic	ref	ref	ref	ref	ref	ref	ref	ref	ref
	Hyperglycemic	1.86	(1.18, 2.92)	0.008	1.39	(0.84, 2.32)	0.203	2.36	(1.46, 3.81)	0.000
Gross motor	FPG (mmol/L)	1.51	(1.13, 2.03)	0.006	1.33	(0.78, 2.28)	0.292	1.51	(1.15, 1.98)	0.003
	Tyg index	1.99	(1.09, 3.65)	0.026	1.20	(0.65, 2.23)	0.555	2.01	(1.11, 3.62)	0.021
	Glycemic status									
	Normalglycemic	ref	ref	ref	ref	ref	ref	ref	ref	ref
	Hyperglycemic	1.64	(1.01, 2.65)	0.045	1.50	(0.88, 2.55)	0.136	1.88	(1.12, 3.15)	0.017
Fine motor	FPG (mmol/L)	1.44	(1.14, 1.84)	0.003	1.24	(0.82, 1.87)	0.299	1.31	(1.04, 1.65)	0.021
	Tyg index	1.31	(0.82, 2.08)	0.259	1.30	(0.82, 2.05)	0.261	1.23	(0.78, 1.94)	0.365
	Glycemic status									
	Normalglycemic	ref	ref	ref	ref	ref	ref	ref	ref	ref
	Hyperglycemic	1.42	(0.99, 2.03)	0.058	1.28	(0.85, 1.94)	0.242	1.62	(1.09, 2.41)	0.017
Cognitive ability	FPG (mmol/L)	1.30	(1.04, 1.62)	0.024	1.46	(1.01, 2.11)	0.044	1.25	(1.01, 1.54)	0.043
	Tyg index	0.96	(0.62, 1.49)	0.872	1.14	(0.75, 1.74)	0.531	0.96	(0.63, 1.47)	0.855
	Glycemic status									
	Normalglycemic	ref	ref	ref	ref	ref	ref	ref	ref	ref
	Hyperglycemic	1.50	(1.08, 2.08)	0.016	1.71	(1.19, 2.45)	0.003	1.53	(1.07, 2.20)	0.020
Language ability	FPG (mmol/L)	1.30	(1.00, 1.69)	0.049	1.10	(0.71, 1.7)	0.676	1.31	(1.03, 1.66)	0.030
	Tyg index	1.24	(0.77, 2.01)	0.371	1.17	(0.73, 1.89)	0.510	1.27	(0.79, 2.02)	0.322
	Glycemic status									

	Normalglycemic	ref	ref	ref	ref	ref	ref	ref	ref	ref
	Hyperglycemic	1.32	(0.90, 1.91)	0.151	1.07	(0.69, 1.67)	0.760	1.53	(1.01, 2.32)	0.046
Social behavior	FPG (mmol/L)	1.35	(1.07, 1.72)	0.012	1.38	(0.94, 2.03)	0.104	1.27	(1.02, 1.60)	0.035
	Tyg index	1.03	(0.66, 1.62)	0.888	1.12	(0.72, 1.75)	0.620	1.02	(0.66, 1.59)	0.920
	Glycemic status									
	Normalglycemic	ref	ref	ref	ref	ref	ref	ref	ref	ref
	Hyperglycemic	1.34	(0.94, 1.89)	0.102	1.07	(0.71, 1.62)	0.745	1.76	(1.20, 2.57)	0.004

DD: developmental dalay; FPG: fasting plasma glucose; TyG: triglyceride glucose; Hyperglycemia was defined as the FPG >5.1 mmol/L; aOR was adjusted by the covariables including maternal age, educational level, occupation, parity, pre-pregnancy BMI, folate intake, season of conception, sex of the offspring, gestational age, birthweight, and age of the offspring.

sTable 10. The associations between glycemia indicators in women with regular folate intake and neurodevelopment quotient

Neurodevelopment	Glycemia indicator	Full-term pregnancy			Early pregnacy			Late pregnancy		
		aBeta	95% CI	P	aBeta	95% CI	P	aBeta	95% CI	P
Overall development	FPG (mmol/L)	-1.44	(-2.17, -0.71)	<0.001	-1.64	(-2.85, -0.43)	0.008	-1.04	(-1.72, -0.37)	0.002
	Tyg index	-1.23	(-2.48, 0.02)	0.055	-1.22	(-2.45, 0.00)	0.051	-1.05	(-2.27, 0.17)	0.092
	Glycemic status									
	Normalglycemic	ref	ref	ref	ref	ref	ref	ref	ref	ref
	Hyperglycemic	-1.53	(-2.51, -0.56)	0.002	-1.09	(-2.23, 0.05)	0.061	-1.66	(-2.79, -0.53)	0.004
Gross motor	FPG (mmol/L)	-0.41	(-1.24, 0.41)	0.327	-1.34	(-2.70, 0.03)	0.055	-0.18	(-0.94, 0.58)	0.648
	Tyg index	-0.49	(-1.9, 0.92)	0.493	-0.62	(-2.00, 0.76)	0.378	-0.36	(-1.73, 1.02)	0.613
	Glycemic status									
	Normalglycemic	ref	ref	ref	ref	ref	ref	ref	ref	ref
	Hyperglycemic	-0.91	(-2.01, 0.19)	0.104	-1.17	(-2.45, 0.11)	0.073	-0.58	(-1.86, 0.69)	0.369
Fine motor	FPG (mmol/L)	-1.44	(-2.26, -0.61)	0.001	-0.91	(-2.27, 0.46)	0.193	-1.09	(-1.85, -0.33)	0.005
	Tyg index	-1.04	(-2.45, 0.38)	0.150	-0.94	(-2.32, 0.45)	0.185	-0.89	(-2.27, 0.49)	0.205
	Glycemic status									
	Normalglycemic	ref	ref	ref	ref	ref	ref	ref	ref	ref
	Hyperglycemic	-1.19	(-2.29, -0.08)	0.035	-0.60	(-1.89, 0.69)	0.360	-1.74	(-3.02, -0.47)	0.007
Cognitive ability	FPG (mmol/L)	-1.63	(-2.77, -0.49)	0.005	-2.61	(-4.49, -0.72)	0.007	-0.97	(-2.02, 0.09)	0.072
	Tyg index	-0.82	(-2.77, 1.13)	0.411	-1.03	(-2.95, 0.88)	0.290	-0.51	(-2.41, 1.40)	0.603
	Glycemic status									
	Normalglycemic	ref	ref	ref	ref	ref	ref	ref	ref	ref
	Hyperglycemic	-1.63	(-3.15, -0.10)	0.037	-2.01	(-3.79, -0.24)	0.026	-1.18	(-2.95, 0.58)	0.189
Language ability	FPG (mmol/L)	-1.46	(-2.44, -0.48)	0.003	-1.85	(-3.47, -0.23)	0.025	-1.07	(-1.97, -0.16)	0.021
	Tyg index	-1.26	(-2.94, 0.42)	0.142	-1.33	(-2.98, 0.31)	0.112	-1.09	(-2.72, 0.55)	0.193
	Glycemic status									

	Normalglycemic	ref	ref	ref	ref	ref	ref	ref	ref	ref
	Hyperglycemic	-1.63	(-2.94, -0.32)	0.015	-1.19	(-2.72, 0.34)	0.126	-1.81	(-3.32, -0.29)	0.019
Social behavior	FPG (mmol/L)	-2.27	(-3.44, -1.10)	<0.001	-1.48	(-3.42, 0.45)	0.134	-1.92	(-3.00, -0.84)	<0.001
	Tyg index	-2.53	(-4.54, -0.53)	0.013	-2.20	(-4.16, -0.23)	0.028	-2.41	(-4.36, -0.46)	0.016
	Glycemic status									
	Normalglycemic	ref	ref	ref	ref	ref	ref	ref	ref	ref
	Hyperglycemic	-2.32	(-3.88, -0.75)	0.004	-0.49	(-2.31, 1.34)	0.602	-2.99	(-4.80, -1.18)	0.001

FPG: fasting plasma glucose; TyG: triglyceride glucose; Hyperglycemia was defined as the FPG >5.1 mmol/L; aBeta was adjusted by the covariables including maternal age, educational level, occupation, parity, pre-pregnancy BMI, folate intake, season of conception, sex of the offspring, gestational age, birthweight, and age of the offspring.

sTable 11. The associations between glycemia indicators in women with regular filate intake and the risk of DD

Neurodevelopment	Glycemia indicator	Full-term pregnancy			Early pregnancy			Late pregnancy		
		aOR	95% CI	P	aOR	95% CI	P	aOR	95% CI	P
Overall development	FPG (mmol/L)	1.43	(1.11, 1.85)	0.006	1.32	(0.8, 2.18)	0.275	1.37	(1.08, 1.75)	0.011
	Tyg index	1.39	(0.77, 2.49)	0.274	1.49	(0.84, 2.63)	0.176	1.36	(0.77, 2.41)	0.292
	Glycemic status									
	Normalglycemic	ref	ref	ref	ref	ref	ref	ref	ref	ref
	Hyperglycemic	1.73	(1.1, 2.72)	0.018	1.30	(0.78, 2.15)	0.311	2.25	(1.39, 3.62)	0.001
Gross motor	FPG (mmol/L)	1.11	(0.81, 1.54)	0.515	1.31	(0.74, 2.3)	0.351	1.10	(0.81, 1.49)	0.551
	Tyg index	1.33	(0.72, 2.46)	0.364	0.96	(0.52, 1.77)	0.899	1.31	(0.72, 2.38)	0.384
	Glycemic status									
	Normalglycemic	ref	ref	ref	ref	ref	ref	ref	ref	ref
	Hyperglycemic	1.47	(0.91, 2.38)	0.112	1.55	(0.92, 2.59)	0.098	1.37	(0.8, 2.34)	0.249
Fine motor	FPG (mmol/L)	1.43	(1.15, 1.78)	0.001	1.30	(0.86, 1.96)	0.212	1.29	(1.05, 1.6)	0.017
	Tyg index	1.30	(0.82, 2.05)	0.259	1.48	(0.95, 2.31)	0.085	1.21	(0.77, 1.88)	0.408
	Glycemic status									
	Normalglycemic	ref	ref	ref	ref	ref	ref	ref	ref	ref
	Hyperglycemic	1.54	(1.08, 2.2)	0.017	1.32	(0.89, 1.97)	0.169	1.62	(1.09, 2.39)	0.016
Cognitive ability	FPG (mmol/L)	1.23	(1, 1.52)	0.052	1.42	(0.98, 2.07)	0.064	1.18	(0.97, 1.44)	0.102
	Tyg index	0.96	(0.63, 1.48)	0.867	1.09	(0.72, 1.64)	0.700	0.95	(0.62, 1.45)	0.814
	Glycemic status									
	Normalglycemic	ref	ref	ref	ref	ref	ref	ref	ref	ref
	Hyperglycemic	1.42	(1.03, 1.97)	0.035	1.59	(1.11, 2.27)	0.011	1.44	(1, 2.06)	0.047
Language ability	FPG (mmol/L)	1.29	(1.02, 1.64)	0.033	1.11	(0.72, 1.73)	0.635	1.28	(1.02, 1.59)	0.030
	Tyg index	1.27	(0.8, 2.04)	0.313	1.35	(0.85, 2.15)	0.200	1.28	(0.81, 2.02)	0.298
	Glycemic status									

	Normalglycemic	ref	ref	ref	ref	ref	ref	ref	ref	ref
	Hyperglycemic	1.22	(0.84, 1.78)	0.288	1.01	(0.66, 1.57)	0.949	1.44	(0.95, 2.17)	0.086
Social behavior	FPG (mmol/L)	1.39	(1.13, 1.73)	0.002	1.35	(0.91, 2.01)	0.133	1.30	(1.06, 1.6)	0.011
	Tyg index	1.18	(0.76, 1.85)	0.462	1.15	(0.74, 1.79)	0.532	1.15	(0.74, 1.78)	0.527
	Glycemic status									
	Normalglycemic	ref	ref	ref	ref	ref	ref	ref	ref	ref
	Hyperglycemic	1.34	(0.94, 1.89)	0.102	1.07	(0.71, 1.62)	0.745	1.76	(1.20, 2.57)	0.004

DD: developmental dalay; FPG: fasting plasma glucose; TyG: triglyceride glucose; Hyperglycemia was defined as the FPG >5.1 mmol/L; aOR was adjusted by the covariables including maternal age, educational level, occupation, parity, pre-pregnancy BMI, folate intake, season of conception, sex of the offspring, gestational age, birthweight, and age of the offspring.

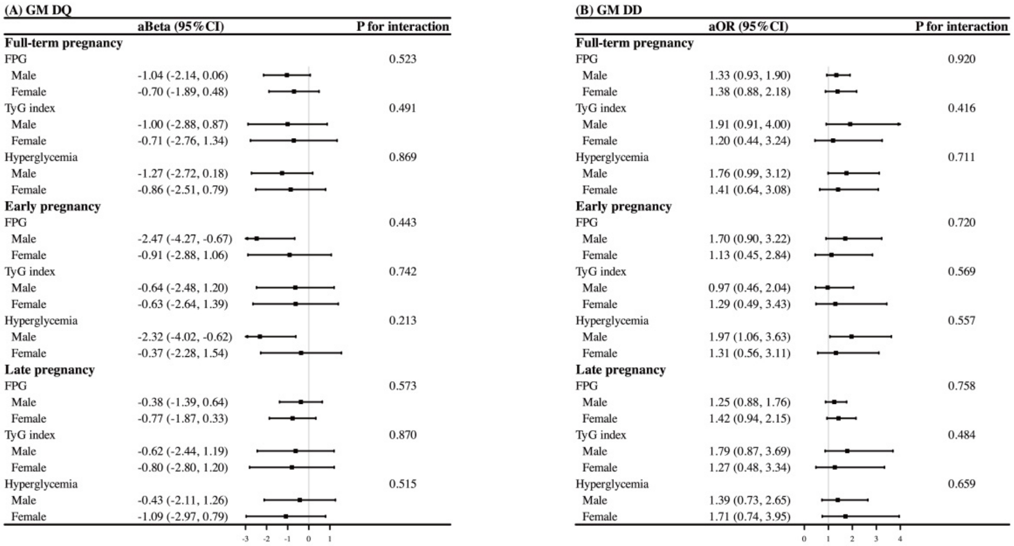

Figure S1. The forestplot for the sex-specific effects of glycemia indicators on the development of gross motor. A showed the associations between glycemia indicators and DQ of gross motor among boys and girls. B presented the assocaitions between glycemia indicators and DD of gross motor among boys and girls.

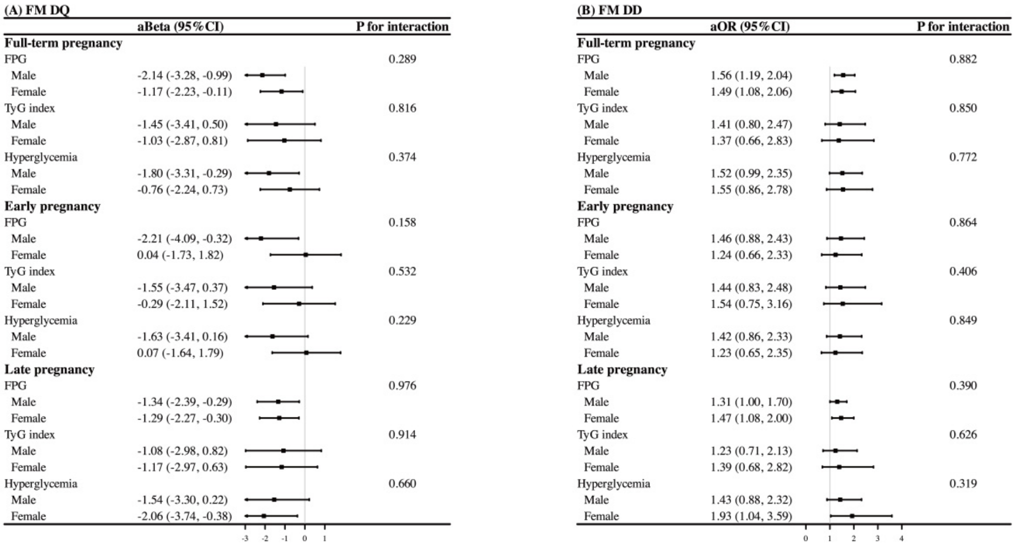

Figure S2. The forestplot for the sex-specific effects of glycemia indicators on the development of fine motor. A showed the associations between glycemia indicators and DQ of fine motor among boys and girls. B presented the assocaitions between glycemia indicators and DD of fine motors among boys and girls.

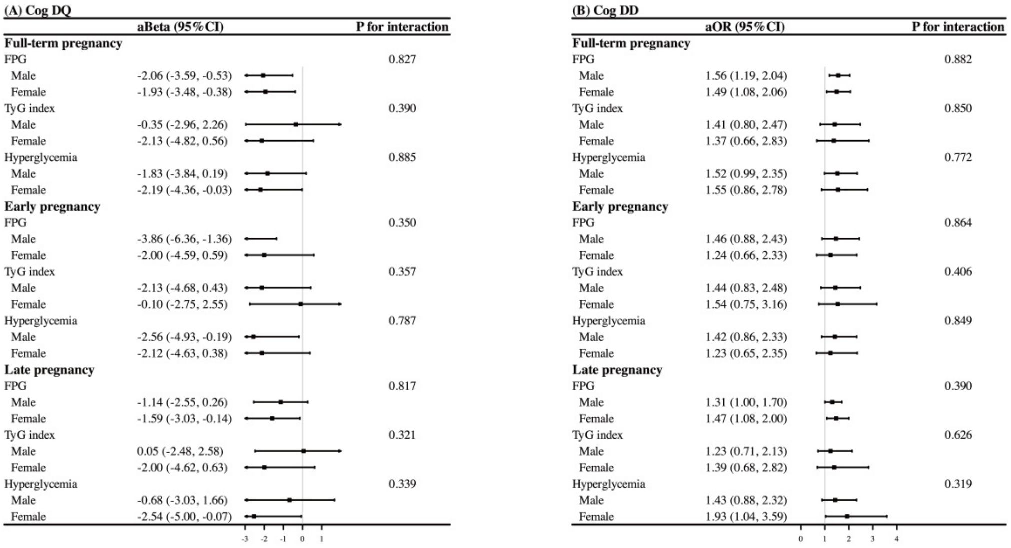

Figure S3. The forestplot for the sex-specific effects of glycemia indicators on the development of cognitive ability. A showed the associations between glycemia indicators and DQ of cognitive ability among boys and girls. B presented the assocaitions between glycemia indicators and DD of cognitive ability among boys and girls.

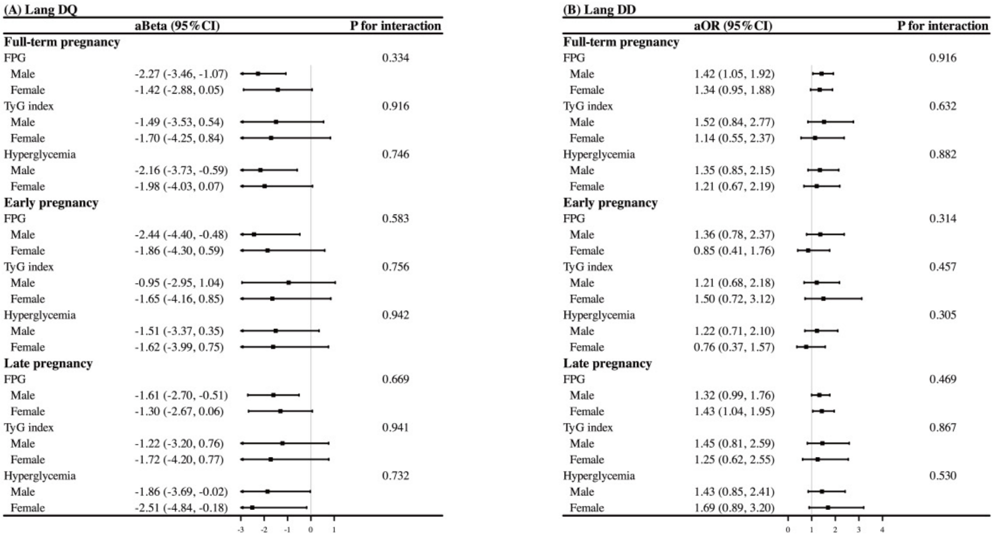

Figure S4. The forestplot for the sex-specific effects of glycemia indicators on the development of language ability. A showed the associations between glycemia indicators and DQ of language ability among boys and girls. B presented the assocaitions between glycemia indicators and DD of language ability among boys and girls.

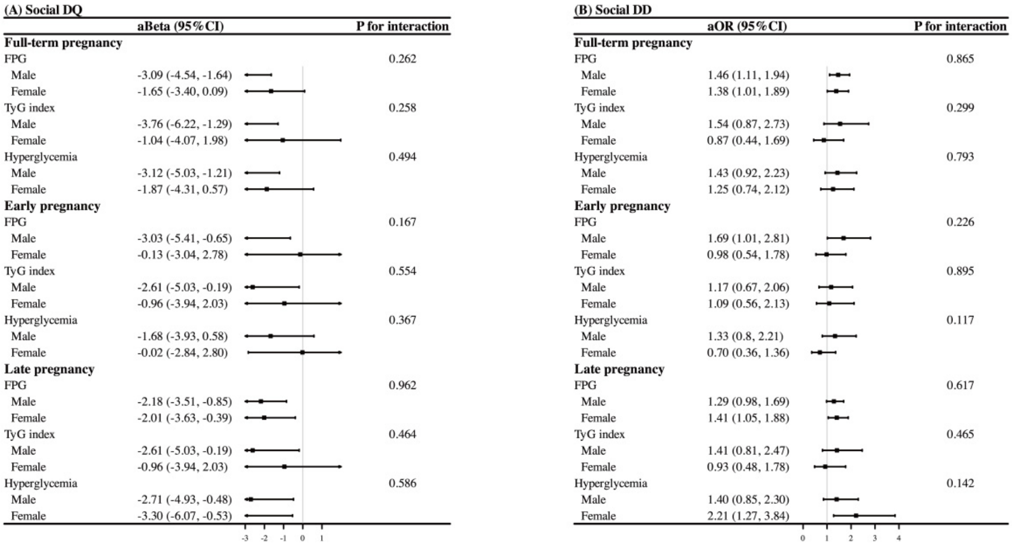

Figure S5. The forestplot for the sex-specific effects of glycemia indicators on the development of social behavior. A showed the associations between glycemia indicators and DQ of social behavior among boys and girls. B presented the assocaitions between glycemia indicators and DD of social behavior among boys and girls.
